# Correlates of serum holo-Transcobalamin in the elderly general population

**DOI:** 10.1007/s00394-025-03789-5

**Published:** 2025-08-31

**Authors:** Paula Stürmer, Eike Andreas Strathmann, Tatjana Patricia Liedtke, Cara Övermöhle, Gerald Rimbach, Katharina Susanne Weber, Wolfgang Lieb

**Affiliations:** 1 Institute of Epidemiology, Kiel University, Niemannsweg 11, 24105 Kiel, Germany; 2 Institute of Human Nutrition and Food Science, Kiel University, Hermann-Rodewald-Straße 6, 24118 Kiel, Germany

**Keywords:** Holo-Transcobalamin, Vitamin B12, General population, Cross-sectional correlates, Stepwise linear regression, Animal derived food products

## Abstract

**Background:**

Holo-Transcobalamin (holo-TC) is the biologically active form of vitamin B12, an essential vitamin that is only contained in animal derived food products. While severe vitamin B12 deficiency has well known clinical consequences, the relation of holo-TC within the physiological range to various metabolic and lifestyle parameters in the general population is largely unknown.

**Objective:**

We aimed to identify cross-sectional metabolic, lifestyle, and dietary correlates of serum holo-TC in a population-based sample.

**Methods:**

Serum holo-TC was measured by electro-chemiluminescence in n = 788 individuals (44.0% females, age 61.3 [52.8; 70.2] years). Significant metabolic and lifestyle correlates of serum holo-TC were identified in a backward selection process and included in a final linear regression model. We used a validated Food Frequency Questionnaire to determine daily dietary intake of dairy products, eggs, fish and seafood, and meat and meat products. Associations of food group intake with holo-TC were tested in multivariable-adjusted analyses, both continuously by linear regression models and across holo-TC tertiles by ANCOVA.

**Results:**

Median serum holo-TC was measured at 83.6 [66.9; 115.2] pmol/L. We identified a 10% increase in alanine aminotransferase, total cholesterol, diastolic blood pressure, or alcohol consumption to confer a 2.03% [1.46; 2.60], 4.58% [1.79; 7.45], −2.66% [−4.78; −0.48], or −0.25% [−0.45; −0.05] change in serum holo-TC, respectively. Compared to no supplementation, vitamin B complex supplementation conferred 19.59% [9.72; 30.34] and females compared to males showed 11.55% [5.38; 18.08] higher serum holo-TC levels. Furthermore, a higher consumption of dairy products (in males and females) and fish and seafood (in males) was associated with significantly higher levels of serum holo-TC.

**Conclusions:**

Vitamin B complex supplementation, dairy products and fish and seafood were identified as major sources of vitamin B12 uptake being reflected in serum holo-TC. Furthermore, sex, total cholesterol, a biomarker of liver function, diastolic blood pressure, and alcohol consumption were associated with serum holo-TC in the northern German population.

**Supplementary Information:**

The online version contains supplementary material available at 10.1007/s00394-025-03789-5.

## Introduction

Vitamin B12 is a nutrient essential in human metabolism that is synthesized by certain microorganisms and that is only contained in animal derived food products [1, 2]. Meat, dairy products, fish, and eggs are considered important sources of bioavailable vitamin B12 [3]. Observational studies reported that intake of these food groups correlated with the levels of biomarkers of vitamin B12 status [4–6]. After dietary ingestion and absorption in the small intestine, vitamin B12 in the circulation is bound to either haptocorrin or transcobalamin [7]. The latter carries only a small proportion of vitamin B12, however, the complex constitutes the biologically active form of this vitamin, called holo-Transcobalamin (holo-TC), as only this complex is available for cell uptake [8, 9]. Holo-TC is, therefore, considered an early marker for vitamin B12 deficiency and is likely suitable to monitor the vitamin B12 status of populations [9].

Vitamin B12 is an essential cofactor for two central metabolic pathways, namely the re-methylation of homocysteine to methionine and the conversion of methylmalonyl-CoA to succinyl-CoA [10, 11]. As such, vitamin B12 is required for erythropoesis [12] and the development and maintenance of the neurological system [11], amongst others. Consequently, functional vitamin B12 deficiency may lead to the development of megaloblastic anemia and central and peripheral neurological manifestations [13]. In contrast to the adverse health outcomes associated with low vitamin B12 status, elevated circulating vitamin B12 has also been associated with an increased risk for certain cancers [14], although it remains unknown whether high circulating vitamin B12 levels are a cause or a consequence of certain forms of cancer.

Despite these reports on the consequences of very low or high levels of this vitamin, little is known about whether variation of vitamin B12 status is related to parameters of metabolic health and lifestyle. In a sample from the National Health and Nutrition Examination Survey (NHANES), serum vitamin B12 was directly associated with age and supplement use and indirectly with smoking and body mass index (BMI), while other lifestyle and sociodemographic variables were not correlated with this vitamin [15]. In a population-based sample from the Netherlands, higher concentrations of biomarkers of liver function were associated with higher plasma concentrations of vitamin B12 [16], while no such association was seen in individuals with cardiometabolic risk factors [17]. No association between serum vitamin B12 and the lipoprotein profile was observed, neither in a clinical sample [18], nor in a subsample from NHANES [19]. In contrast, higher creatinine levels, indicating impaired kidney function, have been shown to be related to higher circulating vitamin B12 [20]. Conceptually, there are different biomarkers to assess an individuals’ vitamin B12 status, including total cobalamin (usually referred to as vitamin B12) and its biologically active form, holo-TC. Compared to vitamin B12 in the circulation, even less is known about holo-TC and its correlates in human metabolism. A direct association with creatinine was reported [10, 20], while in another study age, education level, and BMI were identified as direct correlates of serum holo-TC [21]. However, to our knowledge, metabolic and lifestyle correlates of serum holo-TC have not been investigated systematically in a sample from the general population, thus far. Therefore, we aimed to identify cross-sectional metabolic and lifestyle correlates of serum holo-TC in an elderly population-based sample from the north of Germany. Furthermore, as holo-TC levels are known to be influenced by dietary vitamin B12 intake [4, 22], we assessed the relation between serum holo-TC and the intake of animal derived food products.

## Methods

### Study sample

**Fig. 1:**
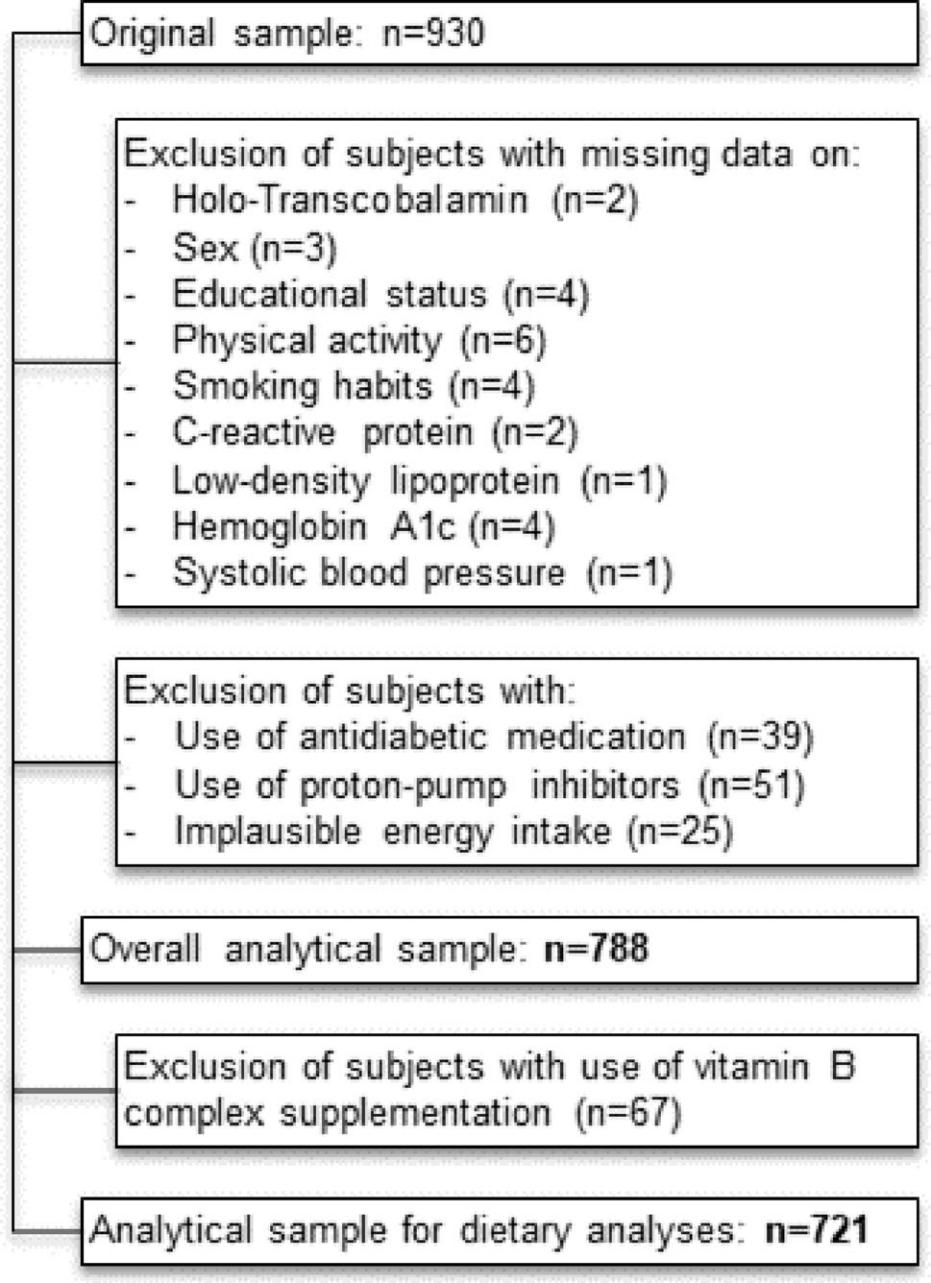
Flowchart of participants of the original study sample being eligible for the current analyses

The current analyses were conducted in a sub-sample of the so called “popgen controls”, a community-based sample that was initially recruited as a reference sample for genetic case–control analyses by the popgen Biobank in Kiel, Germany [23]. Between 2005 and 2007, n = 1316 individuals from population registries and from the blood donation center of the University Hospital in Kiel were recruited. Between 2010 and 2012, n = 930 participants attended a second examination cycle. Data collected at this examination point were used for the current analyses. Examinations included the collection of biomaterials, a physical examination by trained personnel, as well as standardized questionnaires on sociodemographic and lifestyle factors, such as dietary habits, educational status (≤ 9 years, 10 years, ≥ 11 years), smoking habits (never [≤ 3 months smoke duration], former [≥ 3 months smoke duration in the past], and current smokers), and vitamin B complex supplementation (yes *vs.* no). We excluded individuals with missing information on sex, educational status, smoking habits, physical activity, systolic blood pressure, C-reactive protein (CRP), low-density lipoprotein cholesterol (LDL cholesterol), hemoglobin A1c (HbA1c), and holo-TC, as well as those with implausible energy intake (< 500 or > 3500 kcal/d for females, < 800 or > 4000 kcal/d for males [24]). As certain medications can influence the vitamin B12 metabolism [25], we further excluded individuals with self-reported use of proton-pump inhibitors or antidiabetic medication. For dietary analyses, we also excluded individuals who reported to use vitamin B complex supplementation. The final overall analytical sample comprised n = 788 individuals and the sample for dietary analyses comprised n = 721 individuals (Fig. 1). The study adhered to the Declaration of Helsinki and was approved by the ethical review board of the Medical Faculty of the Kiel University (project identification code A 156/03; P2N reference numbers 2022-041 and 2023-026). Written informed consent was obtained from all participants.

### Clinical evaluation

Trained personnel performed anthropometric measurements according to standard operation procedures. We calculated BMI as body weight [kg] divided by height [m] squared. With a sphygmomanometer, blood pressure was measured in duplicate with a two min interval between measurements and an initial resting period of at least five min before the first measurement. For the present analyses, the arithmetic mean of both blood pressure measurements was used. Systolic blood pressure ≥ 140 mmHg or diastolic blood pressure ≥ 90 mmHg or use of antihypertensive medication were defined as hypertension. Using standardized self-administered questionnaires, participants reported their weekly hours spent in physical activity (stairs climbed per day, walking, cycling, gardening, housework, home repair, sports) in the previous 12 months [26]. By summing up the Metabolic Equivalent of Task (MET) values assigned to each activity [27, 28], we calculated physical activity in MET-h/week.

### Laboratory analyses

For biochemical analyses, whole-blood, serum, and lithium heparin plasma samples were drawn from participants in a sitting position. In unfrozen plasma samples, concentrations of HbA1c, CRP, creatinine, liver enzymes (alanine aminotransferase (ALAT), aspartate aminotransferase (ASAT), γ-glutamyl transferase (γ-GT)), and blood lipids (high-density lipoprotein (HDL), LDL, and total cholesterol, triglycerides) were measured on the day of sample collection. HbA1c was measured by high performance liquid chromatography, while for CRP immunoturbidimetry was applied. Liver enzymes, creatinine, and lipoproteins were measured photometrically. For triglycerides, enzymatic colorimetry was used. Individuals with CRP values below the detection limit of 0.9 mg/L were assigned a value of 0.8 mg/L. Biosamples not used for immediate laboratory analyses were stored at − 80 °C until further analyses.

### Measurement of holo-Transcobalamin

Holo-TC was measured in serum samples that had previously been stored at − 80 °C applying electro-chemiluminescence on a cobas® 8000 modular analyzer (Active B12 Elecsys cobas, Roche Diagnostics GmbH, Penzberg, Germany). The combined measurement uncertainty amounted to 9.36%. Individuals with holo-TC values above a limit of 150 pmol/L were assigned values of 151 pmol/L.

### Dietary assessment

By use of a validated, self-administered, semi-quantitative Food Frequency Questionnaire (FFQ) [29], participants reported on their habitual dietary intake in the previous 12 months. The frequency of consumption of 112 foods and beverages was then used to estimate micro- and macronutrient content as well as daily energy intake using the German Food Code and Nutrient Database (II.3) [30]. Items from the questionnaire considered in the dietary analysis were intake in grams per day of dairy products, eggs, fish and seafood, and meat and meat products.

### Statistical analyses

Continuous variables are reported in median and interquartile range (IQR), categorical variables in absolute numbers and percentages. For two sided tests, *p* < 0.05 was considered statistically significant. We performed all analyses with RStudio (v2023.09.1, R for windows 4.3.1).

#### Metabolic and lifestyle correlates of serum holo-TC

Multivariable-adjusted linear regression models were used to identify metabolic and lifestyle correlates of the outcome variable serum holo-TC. This exploratory approach was chosen to determine parameters that influence serum holo-TC apart from intake of vitamin B12 via animal derived food products. To identify the statistically most strongly associated correlates out of a large set of potential correlates, we applied stepwise backward selection procedures using the R package *MASS* [31]. We used Akaike Information Criterion (AIC) as the selection criterion to identify possible predictors of the outcome variable (serum holo-TC). At each step, variables that increased the AIC of the model were removed, until the combination of predictor variables that produced the lowest model AIC value was determined. The candidate variables for this exploratory approach were selected based on the literature and included: educational status, smoking habits, physical activity, systolic and diastolic blood pressure, CRP, creatinine, HbA1c, blood lipids (LDL, HDL, and total cholesterol, triglycerides), liver enzymes (ALAT, ASAT, γ-GT), BMI, supplementation of vitamin B complex, and alcohol consumption. Sex and age were forced into the model. Prior to analyses, all continuous covariates as well as serum holo-TC concentrations were ln-transformed to meet model assumption of normal distribution. For result presentation, β estimates were re-transformed. We present a final multivariable-adjusted linear regression model including serum holo-TC as the dependent variable and the independent variables sex and age as well as all covariates significant in the backward selection process. In addition to this linear regression model, we performed restricted cubic splines (RCS) analyses in the final model to test for overall and nonlinear associations between continuous correlates and serum holo-TC. The R package *plotRCS* [32] was used to place 3 knots at the 10th, 50th, and 90th percentile, respectively, using the 10th percentile as a reference point.

#### Association of dietary intake of animal derived food products with serum holo-TC

We associated the consumption of dairy products, eggs, fish and seafood, and meat and meat products with serum holo-TC in analyses stratified by sex. Prior to all analyses, food groups were adjusted for energy intake using the residual method and applying a constant based on the mean daily energy intake (for males: 2465.1 kcal/d, for females: 1884.0 kcal/d) [33]. Boxplots were designed using the R package *ggplot2* [34]. First, we tested differences in square-root transformed food group consumption across tertiles of serum holo-TC using ANCOVA to control for the covariates age and total daily energy intake. We used the R package *emmeans* [35] to calculate estimated marginal means provided for food group consumption according to serum holo-TC tertiles. Secondly, we used linear regression models, applying the same adjustment as described above, to test for an overall linear association between food group consumption and serum holo-TC. All continuous variables included in these regression models were ln-transformed prior to analysis to meet model assumptions. Consumption of energy adjusted food groups ≤ 0 g/d were set to 0.1 g/d to allow transformation. Parameter estimates were re-transformed for data presentation.

#### Sensitivity analysis excluding participants with serum holo-TC above 150 pmol/L

Furthermore, we performed a sensitivity analysis for the determination of metabolic and lifestyle correlates of serum holo-TC as well as for its association with food groups of animal origin as described above. In these models, we excluded all individuals with holo-TC concentrations above 150 pmol/L to rule out a possible impact on analyses due to the upper limit of the holo-TC measurement.

## Results

### Characterization of the study sample

The overall analytical sample of this study comprised n = 788 individuals (Fig. 1) with a female share of 44.0% and a median age of 61.3 [52.8; 70.2] years. Serum holo-TC concentrations showed an interquartile range from 66.9 to 115.2 pmol/L in the overall sample, which is within the normal physiological range. A total of n = 52 individuals had serum holo-TC concentrations ≤ 50 pmol/L indicating a possible vitamin B12 deficiency [7]. Individuals with higher serum holo-TC tended to have higher levels of total cholesterol, lower diastolic blood pressure, and a lower daily energy and alcohol intake and were more likely to be female. Furthermore, in tertile 3, 15.2% of the individuals indicated to take vitamin B complex supplementations, whereas in tertile 1 only 3.8% took respective supplements. The characterization of the overall sample and according to tertiles of serum holo-TC concentrations is presented in Table 1. For analyses regarding intake of animal derived food products, the analytical sample comprised n = 721 individuals with a female share of 41.7%, a median age of 61.3 [52.8; 70.2] years and a median serum holo-TC concentration of 81.9 [66.0; 111.0] pmol/L. A basic characterization of this subsample as well as intake of food groups is separately reported for males and females in Supplementary Table 1.

**Table 1:** Characterization of the overall analytical sample and stratified by holo-TC tertiles

	Overall sample (n = 788)	Holo-TC Tertile 1 (n = 263)	Holo-TC Tertile 2 (n = 262)	Holo-TC Tertile 3 (n = 263)
Female sex, n (%)	347 (44.0%)	90 (34.2%)	115 (43.9%)	142 (54.0%)
Age [years]	61.3 [52.8; 70.2]	61.3 [50.3; 70.6]	59.5 [50.6; 68.0]	62.4 [55.8; 71.0]
Holo-Transcobalamin [pmol/L]	83.6 [66.9; 115.2]	60.6 [52.0; 66.8]	83.6 [78.4; 93.4]	133.0 [115.5; 151.0]
Height [cm]	172.0 [164.0; 179.0]	173.5 [166.8; 179.8]	173 [164; 180.5]	169.5 [161.5; 177.0]
Weight [kg]	79.1 [69.5; 89.6]	80.4 [72.0; 89.1]	79.6 [71.4; 91.2]	76.3 [66.6; 88.5]
BMI [kg/m^2^]	26.6 [24.2; 29.4]	26.6 [24.6; 29.2]	26.5 [23.9; 29.4]	26.6 [24.0; 29.6]
ALAT [U/L]	22.0 [17.0; 31.0]	21.0 [16.0; 29.0]	23.0 [17.0; 32.0]	23.0 [18.0; 32.0]
ASAT [U/L]	26.0 [22.0; 30.0]	25.0 [22.0; 29.0]	26.0 [22.0; 31.0]	27.0 [23.0; 31.0]
γ-GT [U/L]	24.0 [17.0; 35.0]	23.0 [17.0; 33.0]	24.0 [17.0; 35.0]	25.0 [18.0; 36.0]
HbA1c [%]	5.6 [5.4; 5.9]	5.6 [5.3; 5.8]	5.5 [5.4; 5.8]	5.7 [5.4; 6.0]
LDL cholesterol [mg/dL]	130.0 [110.0; 151.2]	130.0 [106.5; 150.0]	127.0 [110.0; 153.0]	133.0 [112.5; 156.0]
HDL cholesterol [mg/dL]	64.0 [53.0; 76.0]	61.0 [53.0; 72.5]	64.0 [53.0; 78.0]	65.0 [54.0; 79.0]
Total cholesterol [mg/dL]	220.5 [196.0; 249.0]	214.0 [192.0; 245.0]	218.0 [196.0; 251.0]	226.0 [202.5; 252.0]
Triglycerides [mg/dL]	103.5 [76.0; 137.2]	100.0 [78.0; 132.5]	105.0 [72.0; 138.0]	105.0 [76.0; 140.0]
Creatinine [mg/dL]	0.9 [0.8; 1.0]	0.9 [0.8; 1.0]	0.9 [0.8; 1.0]	0.8 [0.7; 1.0]
CRP [mg/L]	1.2 [0.8; 2.5]	1.2 [0.8; 2.2]	1.1 [0.8; 2.4]	1.3 [0.8; 2.8]
Systolic blood pressure [mmHg]	138.8 [125.0; 150.0]	140.0 [126.5; 150.0]	137.5 [125.0; 150.0]	140.0 [125.0; 150.0]
Diastolic blood pressure [mmHg]	84.2 [80.0; 90.0]	85.0 [80.0; 90.0]	84.5 [80.0; 90.0]	82.5 [80.0; 90.0]
Hypertension, n (%)	487 (61.8%)	98 (37.3%)	106 (40.5%)	97 (36.9%)
Physical Activity [MET-h/week]	90.0 [58.1; 130.1]	88.5 [58.9; 130.9]	90.0 [58.2; 122.5]	90.3 [57.5; 137.7]
Total energy intake [kcal/d]	2115.6 [1760.6; 2591.1]	2233.0 [1829.3; 2675.4]	2144.0 [1776.9; 2594.4]	1969.5 [1716.7; 2393.3]
Alcohol consumption [g/d]	9.0 [3.5; 18.5]	10.1 [4.8; 21.5]	9.8 [3.2; 18.6]	7.8 [2.6; 15.7]
Vitamin B complex supplementation, n (%)	67 (8.5%)	10 (3.8%)	17 (6.5%)	40 (15.2%)
Smoking status				
Never, n (%)	116 (14.7%)	39 (14.8%)	41 (15.6%)	36 (13.7%)
Former, n (%)	333 (42.3%)	112 (42.6%)	113 (43.1%)	108 (41.1%)
Current, n (%)	339 (43.0%)	112 (42.6%)	108 (41.2%)	119 (45.2%)
Education				
≤ 9 years, n (%)	261 (33.1%)	84 (31.9%)	80 (30.5%)	97 (36.9%)
10 years, n (%)	250 (31.7%)	79 (30.0%)	88 (33.6%)	83 (31.6%)
≥ 11 years, n (%)	277 (35.2%)	100 (38.0%)	94 (35.9%)	83 (31.6%)

ALAT, alanine aminotransferase; ASAT, aspartate aminotransferase; BMI, body mass index; CRP, C-reactive protein; γ-GT, gamma-glutamyl transferase; HbA1c, hemoglobin A1c; HDL cholesterol, high-density lipoprotein cholesterol; LDL cholesterol, low-density lipoprotein cholesterol; MET-h, Metabolic Equivalent of Task in hours

Continuous variables are given as median [interquartile range], categorical variables as n (%)

### Metabolic and lifestyle correlates of serum holo-TC

After a backward selection process, the variables displayed in Table 2 were identified as correlates of serum holo-TC. The overall model explained 11.9% of the variance in serum holo-TC. In a linear model including all variables mentioned in Table 2, serum holo-TC was significantly directly associated with ALAT and total cholesterol, while diastolic blood pressure and alcohol consumption were inversely associated with holo-TC. Supplementation of vitamin B complex and sex were identified as strong correlates of serum holo-TC with concentrations being 19.59% [9.72; 30.34] higher in individuals using supplements compared with those who do not and being 11.55% [5.38; 18.08] higher in females than in males.

**Table 2:** Metabolic and lifestyle correlates of serum holo-TC concentrations using a linear regression model and restricted cubic splines in the overall analytical sample (n = 788)

	β [95% CI]	*p*_linear_^d^	*p*_nonlinear_^e^	*p*_overall_^e^
Sex^a^	11.55 [5.38; 18.08]	< 0.001	–	–
Age^b^	0.98 [− 0.04; 2.01]	0.059	0.092	0.273
ALAT^b^	2.03 [1.46; 2.60]	< 0.001	0.471	< 0.001
LDL cholesterol^b^	− 1.42 [− 3.21; 0.40]	0.115	0.772	0.296
Total cholesterol^b^	4.58 [1.79; 7.45]	0.001	0.429	0.004
Diastolic blood pressure^b^	− 2.66 [− 4.78; − 0.48]	0.017	0.372	0.039
Vitamin B complex supplementation^c^	19.59 [9.72; 30.34]	< 0.001	–	–
Alcohol consumption^b^	− 0.25 [− 0.45; − 0.05]	0.014	0.322	0.029

Serum holo-TC and continuous independent traits were ln-transformed prior to analyses and β coefficient estimates were re-transformed for presentation of results. Re-transformed β coefficient estimates are interpreted as follows:

^a^ ß_x_ % change in serum holo-TC for male vs. female sex

^b^ 10% increase in a continuous independent trait translates to ß_x_ % change in serum holo-TC, e.g.: a 10% increase in ALAT [U/L] is associated with an increase of 2.03% [1.46; 2.60] of serum holo-TC

^c^ ß_x_ % change in serum holo-TC for supplementation no vs. yes

^d^ p value from linear regression analysis

^e^ p values obtained by restricted cubic splines with knots placed at the 10th, 50th, and 90th percentile

ALAT, alanine aminotransferase; CI, confidence interval; LDL cholesterol, low-density lipoprotein cholesterol

In a sensitivity analysis excluding n = 101 individuals with holo-TC concentrations above 150 pmol/L, the backward selection process identified a final model that was in essence similar to the one described in the overall sample (Supplementary Table 2). In this model, sex (*p*_linear_ < *0.001*), ALAT (*p*_linear_ < *0.001, p*_overall_ < *0.001*), total cholesterol (*p*_linear_ = *0.001, p*_overall_ = *0.003*), and vitamin B complex supplementation (*p*_linear_ = *0.002*) were still identified as significant direct correlates of serum holo-TC and alcohol consumption as an inverse correlate (*p*_linear_ = *0.026*). Further, in this model, HbA1c was significantly directly associated with serum holo-TC, while diastolic blood pressure was no longer identified as a correlate.

### Association of dietary intake of animal derived food products with serum holo-TC

**Fig. 2:**
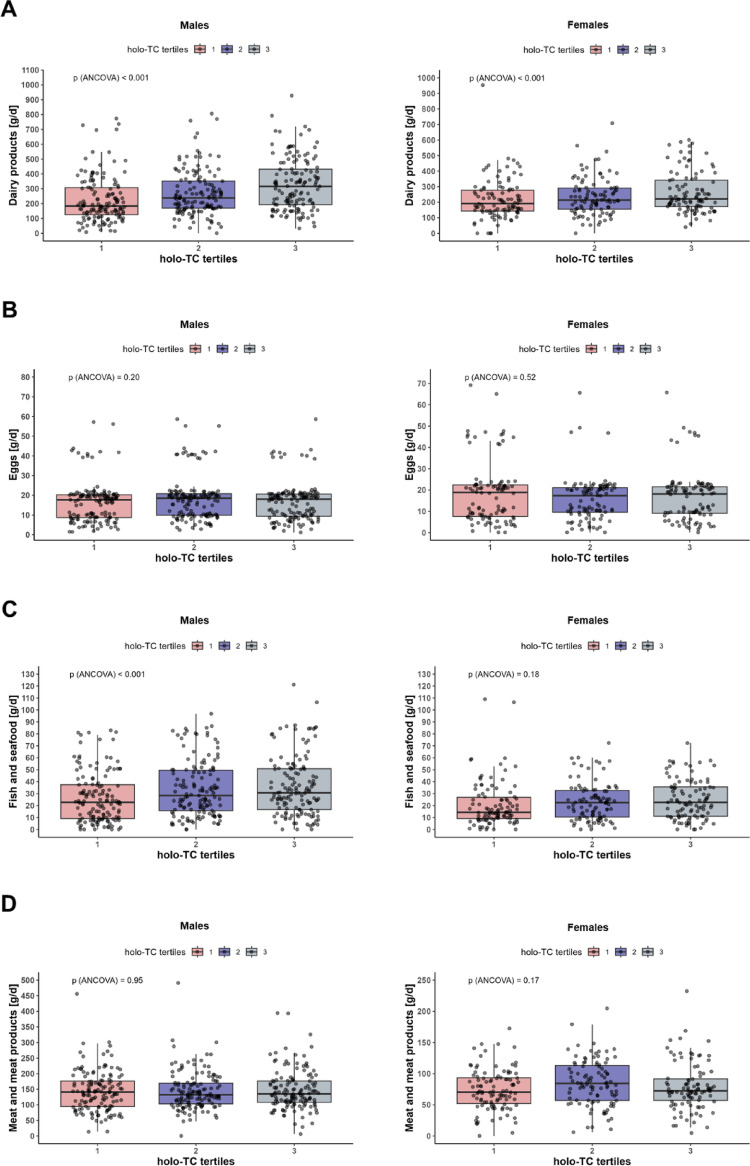
Boxplots of energy adjusted dietary intake of food groups (**A** dairy products, **B** eggs, **C** fish and seafood, **D** meat and meat products) according to tertiles of serum holo-TC of males and females separately after exclusion of users of vitamin B complex supplementation. To test for differences between energy adjusted intakes across holo-TC tertiles, we performed ANCOVA adjusted for age and total daily energy intake. holo-TC, holo-Transcobalamin

As vitamin B12 is an essential vitamin only present in animal derived food products, we aimed to identify the association of four energy-adjusted food groups of animal origin, namely dairy products, eggs, fish and seafood, and meat and meat products, with serum holo-TC, separately for males and females. For males, daily consumption of dairy products and fish and seafood increased significantly with higher serum holo-TC concentrations, both in the linear regression model and when comparing adjusted means across holo-TC tertiles (Table 3, Fig. 2). For females, dairy product consumption also increased significantly with higher serum holo-TC, while for consumption of fish and seafood the linear regression model was only borderline significant (*p* = 0.06). By contrast, the consumption of eggs as well as of meat and meat products was not associated with serum holo-TC, neither in males nor females. In a sensitivity analysis excluding n = 83 participants with serum holo-TC above 150 pmol/L, results did virtually not differ from the primary analysis, except for the association in females between serum holo-TC and fish and seafood consumption now reaching statistical significance in the linear regression model (*p* = 0.01) (Supplementary Table 3).

**Table 3:** Linear association of animal derived food group consumption with serum holo-TC in the analytical sample for dietary analyses (n = 721)

	Linear association of energy-adjusted food groups with serum holo-TC			Estimated marginal means of energy-adjusted food groups by tertiles of serum holo-TC			*p*^e^
	Daily intake	β [95% CI]^c^	*p*	Holo-TC tertile 1^d^	Holo-TC tertile 2	Holo-TC tertile 3	
Males (n = 420)	232.4 [153.5; 361.8]^a^	4.37 [2.58; 6.18]	< 0.001	202.3 [180.0; 225.9]^b^	247.7 [223.1; 273.5]^b^	303.0 [275.5; 331.7]^b^	< 0.001
Females (n = 301)	211.9 [153.8; 293.5]^a^	2.73 [1.25; 4.23]	< 0.001	195.0 [172.6; 218.7]^b^	221.4 [197.3; 246.8]^b^	247.4 [221.9; 274.3]^b^	0.014
Consumption of eggs							
Males (n = 420)	18.0 [9.4; 20.4]^a^	0.35 [−1.75; 2.49]	0.746	14.9 [13.4; 16.5]^b^	16.8 [15.2; 18.5]^b^	15.2 [13.7; 16.9]^b^	0.205
Females (n = 301)	18.2 [8.5; 21.8]^a^	0.26 [−1.34; 1.90]	0.747	16.0 [13.7; 18.4]^b^	14.1 [12.0; 16.4]^b^	14.8 [12.6; 17.1]^b^	0.523
Consumption of fish and seafood							
Males (n = 420)	27.2 [13.2; 47.3]^a^	2.40 [1.21; 3.61]	< 0.001	22.1 [19.0; 25.5]^b^	29.9 [26.3; 33.8]^b^	31.6 [27.7; 35.6]^b^	< 0.001
Females (n = 301)	19.4 [10.1; 33.4]^a^	1.32 [−0.06; 2.71]	0.060	17.6 [14.7; 20.6]^b^	20.5 [17.4; 23.8]^b^	21.6 [18.4; 25.1]^b^	0.183
Consumption of meat and meat products							
Males (n = 420)	135.7 [103.0; 174.9]^a^	−0.17 [−2.52; 2.25]	0.891	137.1 [127.1; 147.5]^b^	137.8 [127.9; 148.1]^b^	139.5 [129.5; 150.0]^b^	0.945
Females (n = 301)	75.6 [55.2; 100.2]^a^	0.25 [−2.12; 2.68]	0.836	70.9 [64.0; 78.1]^b^	80.4 [73.0; 88.2]^b^	73.1 [66.1; 80.5]^b^	0.170

Analyses adjusted for age and daily total energy intake. Prior to analysis, food groups were adjusted for total daily energy intake by use of residual method with addition of the mean daily energy intake as a constant

To meet model assumptions, traits were square root-transformed prior to ANCOVA. For linear regression models, all continuous variables were ln-transformed prior to analyses. Energy adjusted consumption of food groups ≤ 0 g/d was set to 0.1 g/d to allow transformation

^a^ observed means for energy adjusted food groups in [g/d]

^b^ estimated marginal means for energy adjusted food groups in [g/d] adjusted for age and daily total energy intake

^c^ 50% increase in energy adjusted food group consumption translates to ß_x_ % change in serum holo-TC, e.g. a 50% increase in intake of energy adjusted dairy products [g/d] is associated with a 4.37% [2.58; 6.18] increase in serum holo-TC for males

^d^ Males: tertile 1: n = 140, tertile 2: n = 141, tertile 3: n = 139; Females: tertile 1: n = 102, tertile 2: n = 99, tertile 3: n = 100

^e^ *p* value for comparison of adjusted means for square-root-transformed food groups between tertiles of serum holo-TC by use of ANCOVA

CI, confidence interval; holo-TC, holo-Transcobalamin

## Discussion

In this cross-sectional analysis of an elderly population-based sample from northern Germany comprising n = 788 individuals, we assessed metabolic and lifestyle correlates of serum holo-TC, the biologically active form of vitamin B12, as well as its relation to intake of animal derived food products. We found serum holo-TC levels to be statistically significantly associated with total cholesterol, a parameter of liver function (namely ALAT), diastolic blood pressure, and alcohol consumption, as well as to be higher in individuals using vitamin B complex supplementation and in females. Furthermore, in a subsample excluding users of vitamin B complex supplementation, higher serum holo-TC was seen in males and females who consumed higher amounts of dairy products and in males who consumed higher amounts of fish and seafood, whereas intakes of eggs and meat and meat products were not associated with serum holo-TC.

### Holo-TC as the biologically active form of vitamin B12

Holo-TC is considered a good biomarker for monitoring the vitamin B12 status of populations [9] and to have better diagnostic accuracy in detecting vitamin B12 deficiency than circulating vitamin B12 [10, 20, 36, 37]. According to Hermann and Obeid [7], vitamin B12 deficiency is unlikely when serum holo-TC levels exceed 50 pmol/L. In our study sample, only 6.6% of participants fell below this threshold, whereas the middle 50% (interquartile range) of participants had serum holo-TC levels between 66.9 and 115.2 pmol/L. This indicates an overall good vitamin B12 status in our cohort. However, to conclusively detect functional vitamin B12 deficiency, biomarkers of cellular vitamin B12 status, namely homocysteine and methylmalonic acid, should be additionally considered [7, 9, 38], but were, unfortunately, not available in our cohort. Not surprisingly, regular use of supplements that contain vitamin B12 is associated with higher circulating holo-TC [4, 5, 15, 39, 40], an association that was also observed in our sample.

### Dietary correlates of serum holo-TC

The only natural source of vitamin B12 is animal derived food products. In general, observational studies reported that intake of such food groups correlated well with blood levels of biomarkers of vitamin B12 status, namely total cobalamin (often referred to as vitamin B12), holo-TC, and methylmalonic acid [4–6, 22, 41–43]. As a marker of cellular vitamin B12 deficiency, methylmalonic acid generally correlated inversely with intake of animal derived food products [22], while circulating vitamin B12 and holo-TC mostly showed direct associations [5, 6, 22, 42]. However, the extent to which different animal derived food products correlate with these biomarkers varies across food groups and is not entirely consistent in the published literature.

In our sample, we observed (1) a direct association of intake of both dairy products (in males and females) and fish and seafood (in males) with serum holo-TC, and (2) that consumption of eggs and meat and meat products was not associated with circulating holo-TC. The direct association of vitamin B12 status with intake of dairy products is in line with most previous reports [4–6, 22, 41–43]. In one study (n = 728), however, milk consumption in non-vegetarians was directly associated with serum vitamin B12, but not with serum holo-TC [4]. By contrast, in n = 1266 pregnant women, plasma vitamin B12 as well as holo-TC were directly associated with intake of dairy products [22]. This highlights possible differences when relating food group intake to either circulating vitamin B12 or holo-TC, an observation that cannot be explained by the published literature thus far. However, this should be considered when comparing our results with those previously published as most available data is based on circulating vitamin B12.

With regard to fish and seafood consumption, two studies [6, 22] reported a direct association with vitamin B12 status, consistent with the observations in our sample. However, others showed a direct association with serum vitamin B12 only in individuals with low vitamin B12 status (n = 1230) [41] or reported an inverse association of fish intake and serum holo-TC (n = 728) [4].

Contradictory results on the association of circulating biomarkers of vitamin B12 status were also reported for the consumption of meat, the food group with the highest amounts of bioavailable vitamin B12 [44]. In line with our results, some studies showed no association with circulating vitamin B12 or holo-TC [6, 41], whereas others reported a direct association [4, 22, 42]. Potential explanations for the lack of association between meat consumption and vitamin B12 biomarkers [6, 41] include that (1) meal preparation, e.g. cooking and frying, might lead to destruction and loss of bioavailable vitamin B12 due to severe heat influence and water loss [45, 46] and (2) the relative absorption rate of vitamin B12 decreases with higher vitamin concentrations in the meal consumed due to saturation of vitamin B12 receptors [44]. In our cohort, absolute intake of meat and meat products was considerably larger compared to fish and seafood intake. Therefore, a higher relative uptake of vitamin B12 from fish in small portions than from meat in larger portions could help to explain the different associations between these food groups and serum holo-TC in our sample. However, as the accuracy of estimated daily intake amounts by use of a FFQ is limited [47], this point remains speculative. Interestingly, the direct impact of fish consumption on vitamin B12 status underlines the importance of this dietary component with regard to human health outcomes, especially when compared to a rather detrimental effect of high meat consumption [48].

In contrast to the lack of association between egg consumption and serum holo-TC observed in our cohort, two prior studies reported direct associations of egg consumption with holo-TC levels [4, 22]. However, it has been reported that vitamin B12 from eggs is generally poorly absorbed in comparison to other food groups [3, 49].

The new food-based dietary guidelines recently published by the German Nutrition Society emphasize the regular consumption of fish (1–2 portions/week) and dairy products (2 portions/day), while concomitantly recommending a reduced consumption of meat (max. 300 g/week) and eggs (1 egg/week) [50]. As nutrient goals were one of the key aspects incorporated into these recommendations [51], these food-based dietary guidelines support the results of our analyses, namely that we observed no association between serum holo-TC and consumption of meat or eggs, but a direct association with dairy products and fish and seafood intake.

### Metabolic and lifestyle correlates of serum holo-TC

To the best of our knowledge, this is the first study to report metabolic and lifestyle correlates of serum holo-TC in a population-based sample with adequate vitamin B12 status. We identified total cholesterol, a liver function parameter (ALAT), sex, alcohol consumption, and vitamin B complex supplementation as key correlates of serum holo-TC concentrations. Further, diastolic blood pressure was inversely associated with serum holo-TC in the overall analyses, yet was not identified as a correlate in the sensitivity analysis excluding individuals with holo-TC concentrations > 150 pmol/L, which calls into question the reliability of this finding. Moreover, in a population-based sample from the Netherlands, plasma vitamin B12 concentrations were not associated with diastolic or systolic blood pressure [16]. Age, an essential covariate in epidemiologic analyses, was not associated with serum holo-TC in our sample. This contrasts the results of prior studies. Some studies report a higher vitamin B12 status with increasing age [10, 52], while others report this association to be inverse [53, 54].

Females from our analytical sample showed higher serum holo-TC levels than males, which is in accordance with observations in participants aged 60–79 years from a Swiss study [55]. Likewise, in a subsample from NHANES, elderly men had lower serum vitamin B12 and a higher prevalence of vitamin B12 deficiency than women [56]. Contrastingly, healthy female blood donors had lower holo-TC than men, although this association was restricted to individuals ≤ 45 years [57].

Vitamin B12 is involved in lipid metabolism with a low status of this vitamin being associated with dyslipidemia, mostly manifesting itself in elevated total and LDL cholesterol [58]. In a hepatic cell culture model, low vitamin B12 conditions increased fatty acid synthesis leading to higher intracellular fatty acid concentrations [59]. Consistently, in women, a low vitamin B12 status was associated with higher LDL and total cholesterol [60]. However, other studies could not find such associations [18, 19]. We observed a direct association between total cholesterol and holo-TC, which is in line with some of these reports, while other biomarkers of the lipid metabolism were not statistically significantly associated with serum holo-TC in our sample.

We also observed a direct association between serum holo-TC and ALAT, a biomarker of liver function. Similarly, in a population-based sample from the Netherlands (n = 5571), ALAT showed a direct correlation with plasma vitamin B12 [16]. By contrast, in a small (n = 118) cross-sectional study of adults with cardiometabolic risk factors, ALAT was not associated with vitamin B12 concentrations [17]. In clinical samples of patients with alcohol induced hepatocellular damage, higher levels of liver enzymes, including ALAT, were associated with generally higher serum vitamin B12 as it leaked out of the damaged liver tissue [61]. Furthermore, alcoholism was associated with very high levels of serum vitamin B12 in hospital-treated adults [62]. In contrast, in an overall healthy sample from NHANES, only minimal changes in vitamin B12 concentrations with higher alcohol consumption were seen [15], which is in line with the slight inverse association between serum holo-TC and alcohol consumption in our sample. By contrast, in a population-based sample from Denmark, no association between serum vitamin B12 and alcohol consumption was observed [63]. Based on these observations it could be inferred that high alcohol consumption might have an impact on the vitamin B12 metabolism, while alcohol consumption in moderation, as seen in our sample, may have a negligible association with the vitamin B12 status.

Impaired kidney function as indicated by higher creatinine levels, was identified to increase holo-TC levels [10, 20], possibly due to reduced renal holo-TC filtration [20]. In our analytical sample, serum creatinine was not identified as a correlate of serum holo-TC and, therefore, not included in the final linear regression model. However, creatinine levels were within the normal range in our study sample, which might have impeded a statistically significant association between serum holo-TC and this kidney function parameter. Likewise, in an elderly cohort (n = 1209) with low incidence of increased creatinine, holo-TC was not associated with creatinine [64]. Furthermore, creatinine within the reference range was not suggested as a determinant of holo-TC in healthy adults (n = 500) [57]. Contrastingly, in elderly Swedish men (n = 1010), normal-range creatinine levels showed a weak direct correlation with serum holo-TC, while elevated creatinine showed no association with holo-TC [65].

In general, heterogeneity in study samples and the predominant use of circulating vitamin B12 instead of holo-TC in the literature hinders comparability of our results. Furthermore, metabolic and lifestyle correlates explained only a moderate fraction (11.9%) in the interindividual variation of serum holo-TC levels, indicating that other factors might have a considerable impact on holo-TC levels, including, e.g., diet (in particular dairy products and fish and seafood), genetic variation [66], and the gut microbiota [67].

### Strengths and limitations

Strengths of our analyses include the population-based and well-characterized study sample allowing for consideration of a broad variety of metabolic and lifestyle factors. A limitation of the holo-TC measurement (conducted in a professional laboratory) was that the upper detection limit was at 150 pmol/L, which is in contrast to other studies reporting holo-TC values well above this threshold [4, 36, 68]. Setting all individuals above this limit uniformly to 151 pmol/L possibly weakens the results reported here as we could not account for higher serum holo-TC indicating an even better vitamin B12 status. Still, excluding these individuals in sensitivity analyses did hardly change the presented results. Furthermore, in our analyses we only included serum holo-TC, the biologically active form of vitamin B12, as a biomarker of vitamin B12 status and did not measure biomarkers of cellular vitamin B12 deficiency, namely methylmalonic acid or homocysteine [7, 9, 38], and did not take into account other metabolites closely linked to the vitamin B12 metabolism, such as folate [8], which constitutes an important limitation. Another possible limitation is the ascertainment of dietary intake using an FFQ. An FFQ is an established tool in epidemiological studies to ascertain dietary intake over a longer period of time. However, as such, it is prone to misreporting and the daily dietary intake can only be calculated approximately [47]. In general, the backward selection process applied in our study is a data-driven exploratory approach, and as such, chance findings cannot be ruled out. Finally, the study sample of this cross-sectional study comprised elderly individuals of a specific geographical location in northern Germany, which limits the generalizability of our findings.

## Conclusion

In summary, in a cross-sectional analysis of a population-based sample, we observed higher serum holo-TC in individuals with a high intake of dairy products and fish and seafood, reflecting a diet rich in bioavailable vitamin B12, as well as with use of vitamin B complex supplements. Furthermore, we observed total cholesterol, ALAT, diastolic blood pressure, and alcohol consumption to be associated with serum holo-TC and serum holo-TC to be higher in female participants. As we are the first to report metabolic correlates of serum holo-TC in the general population there is a need for future studies to confirm the results presented here as well as to elucidate underlying mechanisms.

## Supplementary Information

Below is the link to the electronic supplementary material.

Supplementary Material 1

Supplementary Material 2

## Data Availability

Data described in the article, code book, and analytic code will be made available upon reasonable request through our P2N data application platform (https://portal.popgen.de/).

## Abbreviations

AIC: Akaike information criterion
CI: Confidence interval
FFQ: Food frequency questionnaire
holo-TC: Holo-Transcobalamin
MET: Metabolic equivalent of task
