# Supplementary material for: Correlates of serum holo-Transcobalamin in the elderly general population
Source: Eur J Nutr. 2025 Aug 31;64(6):270. doi: 10.1007/s00394-025-03789-5 (PMC12399718; doi:10.1007/s00394-025-03789-5)
Supplement: Supplementary file 1 — Supplementary Material 1 [file 394_2025_3789_MOESM1_ESM.docx]

Correlates of serum holo-Transcobalamin in the elderly general population

Paula Stürmer^1^, Eike Andreas Strathmann^1^, Tatjana Patricia Liedtke^1^, Cara Övermöhle^1^, Gerald Rimbach^2^, Katharina Susanne Weber^1*^, Wolfgang Lieb^1*^

*^1^* *Institute of Epidemiology, Kiel University, Kiel, Germany; ^2^ Institute of Human Nutrition and Food Science, Kiel University, Kiel, Germany. None of the authors declare a conflict of interest. * denotes equal contribution*

**
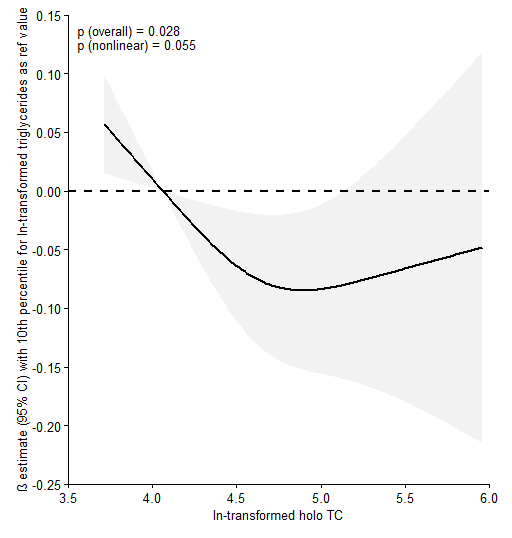
**

**Supplementary Figure 1:** Restricted cubic splines analysis of the association between ln-transformed serum holo-TC and ln-transformed triglycerides.

**Supplementary Table 1:** Metabolic and lifestyle correlates of serum holo-TC concentrations using a linear regression model and restricted cubic splines in n=767 participants after exclusion of those with serum holo-TC above the upper limit (> 150 pmol/L)

|  | **β [95% CI]** | ***p_linear_*^d^** | ***p_nonlinear_*^e^** | ***p_overall_^f^*** |
| --- | --- | --- | --- | --- |
| **Sex^a^** | 9.35 [4.08; 14.89] | **< 0.001** | - | - |
| **Age^b^** | 0.14 [-0.79; 1.08] | 0.770 | 0.466 | 0.524 |
| **ALAT^b^** | 1.03 [0.53; 1.53] | **< 0.001** | 0.070 | **< 0.001** |
| **LDL cholesterol^b^** | -1.42 [-3.05; 0.22] | 0.090 | 0.885 | 0.187 |
| **Total cholesterol^b^** | 3.77 [1.27; 6.32] | **0.003** | 0.993 | **0.029** |
| **Vitamin B complex supplementation^d^** | 17.82 [8.57; 27.85] | **< 0.001** | - | - |
| **Education^c^** | -2.28 [-4.90; 0.41] | 0.096 | - | - |

*Serum holo-TC and continuous independent traits were ln-transformed prior to analyses and β coefficient estimates were re-transformed for presentation of results. Re-transformed β coefficient estimates are interpreted as follows:*

*^a^ ß_x_ % change in serum holo-TC for male vs. female sex*

*^b^ 10% increase in a continuous independent trait translates to ß_x_ % change in serum holo-TC, e.g.: a 10% increase in ALAT [U/L] is associated with an increase of 1.04% [0.53; 1.54] of serum holo-TC.*

*^c^ ß_x_ % change in serum holo-TC for supplementation no vs. yes*

*^d^ ß_x_ % change in serum holo-TC for 9 years education vs. ≥ 10 years
^e^ p value from linear regression analysis*

*^f^ p values obtained by restricted cubic splines with knots placed at the 10^th^, 50^th^, and 90^th^ percentile*

*ALAT, alanine aminotransferase; CI, confidence interval; HbA1c, hemoglobin A1c; LDL cholesterol, low-density lipoprotein cholesterol*

**Supplementary Table 2:** Linear association of animal derived food group consumption with serum holo-TC in a sensitivity analysis (n=767) excluding participants with serum holo-TC above upper test limit (> 150 pmol/L)

|  | Linear association of energy-adjusted food groups with serum holo-TC | | |  | Estimated marginal means of energy-adjusted food groups by tertiles of serum holo-TC | | | | | |  |
| --- | --- | --- | --- | --- | --- | --- | --- | --- | --- | --- | --- |
|  | **Overall sample (n=767)** | **β [95% CI]^c^** | ***p*** |  | **Holo-TC Tertile 1 (n=256)** | | **Holo-TC Tertile 2 (n=255)** | | **Holo-TC Tertile 3 (n=256)** | | ***p^d^*** |
| **Consumption of dairy products** | | | | | | | | | | | |
| **Model 1** | 220.0 [148.2; 327.4]^a^ | 2.53 [1.45; 3.63] | **< 0.001** |  | 201.4 [185.6; 217.9]^b^ | | 242.1 [224.9; 259.9]^b^ | | 258.5 [240.9; 276.8]^b^ | | **< 0.001** |
| **Model 2** | 220.0 [148.8; 327.4]^a^ | 2.49 [1.40; 3.59] | **< 0.001** |  | 201.5 [185.7; 218.0]^b^ | | 241.6 [224.3; 259.5]^b^ | | 258.3 [240.7; 276.6]^b^ | | **< 0.001** |
| **Model 3** | 220.0 [148.8; 327.4]^a^ | 2.40 [1.32; 3.49] | **< 0.001** |  | 205.2 [182.9; 228.6]^b^ | | 245.5 [221.5; 270.7]^b^ | | 261.7 [238.8; 285.8]^b^ | | **< 0.001** |
| **Consumption of eggs** | |  |  |  | |  | |  | |  |  |
| **Model 1** | 17.7 [9.0; 21.4]^a^ | 0.09 [-1.07; 1.26] | 0.881 |  | 15.2 [13.9; 16.6]^b^ | | 16.2 [14.8; 17.5]^b^ | | 15.8 [14.5; 17.1]^b^ | | 0.626 |
| **Model 2** | 17.7 [9.0; 21.4]^a^ | 0.09 [-1.07; 1.26] | 0.873 |  | 15.2 [13.9; 16.6]^b^ | | 16.2 [14.8; 17.6]^b^ | | 15.8 [14.5; 17.1]^b^ | | 0.609 |
| **Model 3** | 17.7 [9.0; 21.4]^a^ | 0.00 [-1.15; 1.17] | 0.993 |  | 16.2 [14.3; 18.2]^b^ | | 17.2 [15.3; 19.2]^b^ | | 16.6 [14.9; 18.4]^b^ | | 0.624 |
| **Consumption of fish and seafood** | | |  |  |  | |  | |  | |  |
| **Model 1** | 23.1 [12.0; 38.1]^a^ | 1.83 [1.11; 2.55] | **< 0.001** |  | 19.2 [17.1; 21.4]^b^ | | 25.5 [23.1; 28.0]^b^ | | 26.6 [24.1; 29.1]^b^ | | **< 0.001** |
| **Model 2** | 23.1 [12.0; 38.1]^a^ | 1.81 [1.08; 2.55] | **< 0.001** |  | 19.2 [17.1; 21.5]^b^ | | 25.2 [22.8; 27.8]^b^ | | 26.4 [24.0; 29.0]^b^ | | **< 0.001** |
| **Model 3** | 23.1 [12.0; 38.1]^a^ | 1.78 [1.05; 2.52] | **< 0.001** |  | 19.5 [16.5; 22.7]^b^ | | 25.5 [22.2; 29.1]^b^ | | 26.7 [23.5; 30.0]^b^ | | **< 0.001** |
| **Consumption of meat and meat products** | | |  |  |  | |  | |  | |  |
| **Model 1** | 111.4 [81.9; 144.2]^a^ | 0.61 [-0.56; 1.80] | 0.307 |  | 104.1 [97.7; 111.7]^b^ | | 110.1 [103.6; 116.8]^b^ | | 108.1 [101.7; 114.6]^b^ | | 0.424 |
| **Model 2** | 111.4 [81.9; 144.2]^a^ | 0.46 [-0.76; 1.69] | 0.462 |  | 104.4 [98.2; 110.8]^b^ | | 108.2 [101.9; 114.7]^b^ | | 107.2 [101.0; 113.6]^b^ | | 0.687 |
| **Model 3** | 111.4 [81.9; 144.2]^a^ | 0.48 [-0.72; 1.71] | 0.432 |  | 101.3 [92.7; 110.2]^b^ | | 105.1 [96.5; 114.1]^b^ | | 104.6 [96.6; 112.8]^b^ | | 0.652 |

*Model 1: adjusted for sex and age. Model 2: additionally, adjusted for daily total energy intake. Model 3: additionally, adjusted for supplementation of vitamin B complex. For all models, food groups were adjusted for total daily energy intake by use of residual method with addition of the mean daily energy intake as a constant
To meet model assumptions, traits were square root-transformed prior to ANCOVA. For linear regression models, all continuous variables were ln-transformed prior to analyses. Energy adjusted consumption of food groups ≤ 0 g/d was set to 0.1g/d to allow transformation.*

*^a^ observed means for energy adjusted food groups in [g/d]*

*^b^ estimated marginal means for energy adjusted food groups in [g/d] adjusted for covariates as specified in the respective models*

*^c^ 50% increase in energy adjusted food group consumption translates to ß_x_ % change in serum holo-TC, e.g. a 50% increase in intake of energy adjusted dairy products [g/d] is associated with a 2.40% [1.32; 3.49] increase in serum holo-TC in model 3. ^d^ p value for comparison of adjusted means for square-root-transformed food groups between tertiles of serum holo-TC by use of ANCOVA
CI, confidence interval; holo-TC, holo-Transcobalamin*
