# Supplementary material for: Correlates of serum holo-Transcobalamin in the elderly general population
Source: Eur J Nutr. 2025 Aug 31;64(6):270. doi: 10.1007/s00394-025-03789-5 (PMC12399718; doi:10.1007/s00394-025-03789-5)
Supplement: Supplementary file 2 — Supplementary Material 2 [file 394_2025_3789_MOESM2_ESM.docx]

Correlates of serum holo-Transcobalamin in the elderly general population

Paula Stürmer^1^, Eike Andreas Strathmann^1^, Tatjana Patricia Liedtke^1^, Cara Övermöhle^1^, Gerald Rimbach^2^, Katharina Susanne Weber^1*^, Wolfgang Lieb^1*^

*^1^* *Institute of Epidemiology, Kiel University, Kiel, Germany; ^2^ Institute of Human Nutrition and Food Science, Kiel University, Kiel, Germany. None of the authors declare a conflict of interest. * denotes equal contribution*

**Supplementary Table 1:** Characterization of the analytical sample for dietary analyses (n=721) stratified by sex

|  | **Males (n=420)** | **Females (n=301)** |
| --- | --- | --- |
| **Age [years]** | 61.1 [53.7; 69.8] | 61.4 [51.3; 71.1] |
| **Holo-Transcobalamin [pmol/L]** | 78.2 [64.2; 105.0] | 90.3 [71.5; 119.0] |
| **BMI [kg/m²]** | 26.8 [25.1; 29.3] | 25.8 [22.9; 29.3] |
| **Physical Activity  [MET-h/week]** | 82.6 [55.2; 125.4] | 96.8 [64.8; 137.3] |
| **Smoking status** |  |  |
| **Never, n (%)** | 64 (15.2%) | 49 (16.3%) |
| **Former, n (%)** | 210 (50.0%) | 91 (30.2%) |
| **Current, n (%)** | 146 (34.8%) | 161 (53.5%) |
| **Education** |  |  |
| **≤ 9 years, n (%)** | 139 (33.1%) | 108 (35.9%) |
| **10 years, n (%)** | 112 (26.7%) | 115 (38.2%) |
| **≥ 11 years, n (%)** | 169 (40.2%) | 78 (25.9%) |
| **Total energy intake [kcal/d]** | 2399.0 [2054.7; 2851.5] | 1829.4 [1589.3; 2106.0] |
| **Alcohol consumption [g/d]** | 12.4 [5.7; 23.2] | 6.2 [2.1; 12.3] |
| **Food group intake [g/d]** |  |  |
| **Vegetables** | 177.8 [145.4; 214.7] | 173.6 [142.1; 211.0] |
| **Fruits** | 165.0 [112.2; 233.1] | 198.0 [144.8; 345.3] |
| **Nuts and seeds** | 2.9 [0.9; 3.4] | 1.3 [0.8; 4.9] |
| **Legumes** | 2.9 [0.9; 3.4] | 1.3 [0.8; 4.9] |
| **Cereal** | 144.5 [119.1; 198] | 121.8 [98.8; 160.1] |
| **Dairy products** | 229.8 [145.7; 376.7] | 207 [146.4; 303.2] |
| **Eggs** | 18.1 [9.0; 20.0] | 19.9 [8.7; 21.1] |
| **Fish and seafood** | 32.2 [16.5; 44.4] | 22.2 [11.8; 35.2] |
| **Meat and meat products** | 135.5 [92.9; 181.0] | 75.7 [52.0; 102.7] |
| **Sugar and confectionary** | 46.5 [32.8; 68.4] | 42.9 [29.0; 56.9] |

*MET-h, Metabolic Equivalent of Task in hours*

*Continuous variables are given as median [interquartile range], categorical variables as n (%).*

**Supplementary Table 2:** Metabolic and lifestyle correlates of serum holo-TC concentrations using a linear regression model and restricted cubic splines in the overall analytical sample (n=687) after exclusion of participants with serum holo-TC above the upper limit (>150 pmol/L)

|  | **β [95% CI]** | ***p_linear_*^d^** | ***p_nonlinear_*^e^** | ***p_overall_^e^*** |
| --- | --- | --- | --- | --- |
| **Sex^a^** | 12.13 [6.50; 18.05] | **< 0.001** | - | - |
| **Age^b^** | 0.03 [-0.97; 1.05] | 0.950 | 0.426 | 0.728 |
| **ALAT^b^** | 1.47 [0.91; 2.04] | **< 0.001** | 0.237 | **< 0.001** |
| **HbA1c^b^** | 3.06 [0.10; 6.12] | **0.043** | **0.038** | **0.017** |
| **Total cholesterol^b^** | 2.31 [1.00; 3.63] | **< 0.001** | 0.798 | **0.003** |
| **Triglycerides** | -0.39 [-0.89; 0.10] | 0.119 | **0.015** | **0.017** |
| **Vitamin B complex supplementation^d^** | 14.5 [4.89; 24.99] | **0.002** | - | - |
| **Alcohol consumption^c^** | -0.01 [-0.02; 0.00] | **0.026** | 0.206 | 0.154 |

*Serum holo-TC and continuous independent traits were ln-transformed prior to analyses and β coefficient estimates were re-transformed for presentation of results. Re-transformed β coefficient estimates are interpreted as follows:*

*^a^ ß_x_ % change in serum holo-TC for male vs. female sex*

*^b^ 10% increase in a continuous independent trait translates to ß_x_ % change in serum holo-TC, e.g.: a 10% increase in ALAT [U/L] is associated with an increase of 1.47% [0.91; 2.04] of serum holo-TC.*

*^c^ ß_x_ % change in serum holo-TC for supplementation no vs. yes*

*^d^ p value from linear regression analysis*

*^e^ p values obtained by restricted cubic splines with knots placed at the 10^th^, 50^th^, and 90^th^ percentile*

*ALAT, alanine aminotransferase; CI, confidence interval; HbA1c, hemoglobin A1c*

**Supplementary Table 3:** Linear association of animal derived food group consumption with serum holo-TC in the analytical sample for dietary analyses (n=638) after exclusion of participants with serum holo-TC above the upper limit (>150 pmol/L)

|  | Linear association of energy-adjusted food groups with serum holo-TC | | |  | Estimated marginal means of energy-adjusted food groups by tertiles of serum holo-TC | | | | | |  |
| --- | --- | --- | --- | --- | --- | --- | --- | --- | --- | --- | --- |
|  | **Daily intake** | **β [95% CI]^c^** | ***p*** |  | **Holo-TC Tertile 1^d^** | | **Holo-TC Tertile 2** | | **Holo-TC Tertile 3** | | ***p^e^*** |
| **Consumption of dairy products** | | | | | | | | | | | |
| **Males (n=375)** | 223.3 [150.2; 354.5]^a^ | 4.21 [2.39; 6.05] | **< 0.001** |  | 199.5 [176.3; 224.1]^b^ | | 247.6 [221.9; 274.7]^b^ | | 285.5 [257.8; 314.7]^b^ | | **< 0.001** |
| **Females (n=263)** | 210.5 [149.5; 286.2]^a^ | 2.14 [0.79; 3.51] | **0.002** |  | 185.7 [162.6; 210.5]^b^ | | 231.2 [204.9; 259.0]^b^ | | 229.9 [203.9; 257.6]^b^ | | **0.020** |
| **Consumption of eggs** | |  |  |  | |  | |  | |  |  |
| **Males (n=375)** | 18.1 [9.6; 20.5]^a^ | 1.81 [-0.08; 3.74] | 0.061 |  | 14.7 [13.1; 16.4]^b^ | | 16.7 [15.0; 18.5]^b^ | | 16.7 [15.0; 18.5]^b^ | | 0.185 |
| **Females (n=263)** | 17.5 [8.4; 21.8]^a^ | -0.46 [-1.96; 1.06] | 0.552 |  | 15.4 [13.1; 17.9]^b^ | | 14.5 [12.2; 17.0]^b^ | | 14.1 [11.8; 16.5]^b^ | | 0.742 |
| **Consumption of fish and seafood** | | |  |  |  | |  | |  | |  |
| **Males (n=375)** | 26.8 [12.4; 45.7]^a^ | 2.05 [0.97; 3.14] | **< 0.001** |  | 21.7 [18.4; 25.3]^b^ | | 28.9 [25.1; 33.0]^b^ | | 30.8 [26.9; 35.0]^b^ | | **0.002** |
| **Females (n=263)** | 18.5 [10.1; 32.4]^a^ | 1.79 [0.43; 3.17] | **0.010** |  | 17.2 [14.2; 20.5]^b^ | | 19.9 [16.7; 23.5]^b^ | | 22.5 [19.0; 26.2]^b^ | | 0.100 |
| **Consumption of meat and meat products** | | |  |  |  | |  | |  | |  |
| **Males (n=375)** | 136.4 [104.9; 175.5]^a^ | 0.46 [-1.68; 2.66] | 0.674 |  | 134.8 [124.4; 145.5]^b^ | | 143.2 [132.6; 154.2]^b^ | | 139.7 [129.2; 150.7]^b^ | | 0.549 |
| **Females (n=263)** | 76.7 [54.7; 99.6]^a^ | -0.47 [-2.70; 1.82] | 0.685 |  | 72.5 [65.2; 80.3]^b^ | | 77.1 [69.4; 85.1]^b^ | | 71.9 [64.5; 79.7]^b^ | | 0.598 |

*Analyses adjusted for age and daily total energy intake. Prior to analysis, food groups were adjusted for total daily energy intake by use of residual method with addition of the mean daily energy intake as a constant.*

*To meet model assumptions, traits were square root-transformed prior to ANCOVA. For linear regression models, all continuous variables were ln-transformed prior to analyses. Energy adjusted consumption of food groups ≤ 0 g/d was set to 0.1g/d to allow transformation.*

*^a^ observed means for energy adjusted food groups in [g/d]*

*^b^ estimated marginal means for energy adjusted food groups in [g/d] adjusted for age and daily total energy intake*

*^c^ 50% increase in energy adjusted food group consumption translates to ß_x_ % change in serum holo-TC, e.g. a 50% increase in intake of energy adjusted dairy products [g/d] is associated with a 4.21 % [2.39; 6.05] increase in serum holo-TC for males.*

*^d^ Males: tertile 1: n=125, tertile 2: n=125, tertile 3: n=125; Females: tertile 1: n=89, tertile 2: n=86, tertile 3: n=88*

*^e^ p value for comparison of adjusted means for square-root-transformed food groups between tertiles of serum holo-TC by use of ANCOVA*

*CI, confidence interval; holo-TC, holo-Transcobalamin*
